# Age-Related Changes in Female Murine Reproductive Mucosa with respect to *γδ* T Cell Presence

**DOI:** 10.1155/2023/3072573

**Published:** 2023-01-23

**Authors:** Katarzyna Skulska, Anna Ewa Kędzierska, Małgorzata Krzyżowska, Grzegorz Chodaczek

**Affiliations:** ^1^Łukasiewicz Research Network-PORT Polish Center for Technology Development, Wroclaw, Poland; ^2^Hirszfeld Institute of Immunology and Experimental Therapy, Polish Academy of Sciences, Wroclaw, Poland; ^3^Military Institute of Hygiene and Epidemiology, Warsaw, Poland

## Abstract

Many studies have demonstrated a general decline and dysregulation in immune functions with age. It is not clear, however, how the aging affects the immune surveillance of the female reproductive tract (FRT) by *γδ* T cells, a unique population of T lymphocytes that was shown to regulate homeostasis of epithelial barriers. First, we analyzed *γδ* T cell presence in FRT in young (2 months) and old (18 months) wild-type (WT) C57BL/6 mice. We did not detect any changes in *γδ* T cell number nor distribution in the vaginas between the age groups, while in uteri, there was a twofold increase in *γδ* T cell number in aged mice. To check if *γδ* T lymphocytes regulate a metabolic and immune status of aging vaginal tissue, we compared the expression of 84 aging-associated genes in young and old WT and *γδ* T-cell-deficient (*Tcrd*^−/−^) mice. We discovered that only the *Ltf* (lactotransferrin) gene was downregulated in old *Tcrd*^−/−^ mice. In both mouse strains, we found similar age-dependent changes in cytokine production upon vaginal inflammation due to Toll-like receptor 9 (TLR9) stimulation with CpG. With age in the vaginas, IL-1*α* and IL-17A levels increased while IL-6, IL-10, MCP-1, and IFN*γ* levels were diminished in response to CpG. Similar trends were observed in uteri. Interestingly, under the inflammatory state, the lack of *γδ* T cells in young individuals enhanced MCP-1 production in the vagina and decreased MCP-1 level in the uterus in old females. Our gene expression data point to an antimicrobial role of *γδ* T lymphocytes. The profile of secreted inflammatory cytokines shifted during aging toward the proinflammatory type, and *γδ* T cells played a modest fine-tuning role in immunoregulation in aged FRT. We believe this work expands our understanding of *γδ* T cell functions and the inflammaging in the murine reproductive epithelia.

## 1. Introduction

In mucosal tissues, intraepithelial T lymphocytes (IELs) and their subset expressing a T cell receptor with *γ* and *δ* chains (*γδ* TCR) are the most important immune cell populations being responsible for tissue homeostasis maintenance and forming the first line of defense against pathogens [1]. The main functions of *γδ* IELs have been recognized through epidermal studies in mice, but their role in the reproductive system remains largely unexplored [1]. The structure of *γδ* TCRs depends on the site of localization. Within the vaginal epithelium prevail IELs with the V*γ*6-V*δ*1 TCR [2]. *Γδ* T cells act as a “bridge” between innate and adaptive immunity. On the one hand, they can directly sense tissue stress and respond rapidly to various forms of cell dysregulation using additional costimulatory signals of self-expressed NKG2D molecule. On the other hand, through the production of chemokines, cytokines, and antigen presentation, they may contribute to the priming of adaptive immune responses by interacting with dendritic cells (DCs), B lymphocytes, or other T cells [3]. It is suggested that *γδ* T cells in the female reproductive tract (FRT) play a role in epithelium self-renewal mechanisms (based on the keratinocyte growth factor (KGF) mRNA synthesis) and in antiviral responses [2, 4–6]. Nonetheless, there is a general agreement in published data that viral infections induce proliferation of vaginal *γδ* T cells but not *αβ* T cells, suggesting TCR-related proliferative response due to environmental stress [4–7].

It is well established that sex hormones affect immune function of the reproductive system during the menstrual cycle; however, relatively, little is known about the immunosenescent changes that occur after menopause [8]. As sex hormone levels drop in aged females, the epithelial barrier becomes thinner with lower regenerative capabilities leading to a higher risk of infections [8]. At the same time in postmenopausal women, changes occur in cell number and distribution of many immune cells migrating to or residing in the reproductive epithelium such as CD4^+^ T lymphocytes, CD8^+^ T lymphocytes, CD11c^+^ DCs, CD1a^+^ antigen-presenting cells (APCs), and macrophages [8]. In result, epithelial cytokine milieu and antimicrobial peptide production are dysregulated, which further increases the infection susceptibility of old individuals. With this in mind, we decided to investigate whether the age affects the number of *γδ* T cells and their distribution. We also compared young and old wild-type C57BL/6J (WT) and *γδ* T-cell-deficient (*Tcrd*^−/−^) female mice and assessed gene expression profile and cytokine production at the steady state and in response to inflammation in order to clarify homeostatic functions of *γδ* T lymphocytes within FRT.

## 2. Materials and Methods

### 2.1. Mice

C57BL/6J and *Tcrd^−/−^* mice [9] were purchased from the Jackson Laboratory (Bar Harbor, ME, USA). Tcrd-H2BeGFP transgenic mice (C57BL/6 background) were a kind gift of Dr. Immo Prinz (Institute of Immunology, Hanover Medical School, Hanover, Germany). Tcrd-H2BeGFP mice are transgenic fluorescent reporter mice enabling the identification of *γδ* T lymphocytes due to an insertion of an expression cassette encoding an internal ribosomal entry site and a fusion of human histone H2B and green fluorescent protein (GFP) in the 3′ untranslated region of the *Tcrd* constant (C) gene [10]. Thus, GFP is expressed in nuclei of *γδ* T cells and labels only this cell population. All mice were housed at the Łukasiewicz Research Network–PORT Polish Center For Technology Development (Łukasiewicz-PORT) in Wroclaw, Poland, in individually ventilated cages in 12 : 12 h light-dark cycle under specific pathogen-free conditions with water and food available *ad libitum*. Young female mice at the age of 2 months were subcutaneously injected with 3 mg of medroxyprogesterone acetate (Depogeston, Biowet Puławy, Puławy, Poland) to induce diestrus. The stage of the estrous cycle was determined by vaginal smears. Females were kept at the Łukasiewicz-PORT's animal facility until they reached 18 months of age. All experiments were in accordance with the Local Ethics Committee for Experiments on Animals at Hirszfeld Institute of Immunology and Experimental Therapy in Wroclaw.

### 2.2. Vaginal Inflammation

Mice were anesthetized with ketamine/xylazine (ketamine 100 mg/kg (Biowet Puławy, Poland) + xylazine 10 mg/kg (Biowet Puławy)) and treated intravaginally with 20 *μ*g ODN 1826 (CpG, Toll-like receptor 9 (TLR9) agonist) (InvivoGen, CA, USA) in 1.5% 2-hydroxyethyl cellulose solution (Sigma-Aldrich, Germany) in a total volume of 20 *μ*l. After administration, the mice were placed in a cage on their backs to avoid leakage of the administered solutions. Twenty four hours after the induction of inflammation with CpG, the vaginas and uteri were processed for cytokine analysis.

### 2.3. Isolation of Female Reproductive Tract (FRT) Cells

The vaginas were isolated and analyzed using a previously developed protocol [11]. Young (2 months) and old (18 months) C57BL/6J mice were euthanized with cervical dislocation. Reproductive tracts were dissected, divided into the vagina and uterus (cervix and uterine horns), and placed on ice in PBS (Thermo Fisher Scientific, Waltham, MA, USA). The vagina and uterus were minced separately into small fragments in a 2 ml Eppendorf tube using curved dissection scissors and incubated with 1 mg/ml Liberase TL (Roche, Mannheim, Germany; 5.2 U/mg) in Hank's Balanced Salt Solution (HBSS, Thermo Fisher Scientific) supplemented with 30 *μ*g/ml DNase I (grade II, from bovine pancreas, Roche) at 37°C for 1 h with shaking (Eppendorf ThermoMixer C, 800 rpm). After digestion, tissues were passed through a 40 *μ*m cell strainer (Corning, Corning, NY, USA), placed in a 50 ml conical tube, washed in PBS, and centrifuged at 300 × g for 5 min at 4°C.

### 2.4. Flow Cytometry Analysis

The FRT-isolated cells from Tcrd-H2BeGFP mice were resuspended in 3 ml of RPMI 1640 (Sigma-Aldrich, Saint Louis, MO, USA), transferred to 3 ml of Lympholyte-M Cell Separation Media (1.0875 ± 0.001 g/ml, Cedarlane Laboratories, Burlington, NC, USA) in a 15 ml conical tube and centrifuged at 300 × g for 20 min (accel 2, brake 1, Eppendorf 5810R Series Centrifuge, Rotor A-4-62) at room temperature (RT). The cells at the interface were carefully removed using a serological pipette, transferred into a new tube, and washed with PBS. The cell suspensions were incubated with Fixable Viability Dye eFluor 780 (1 : 1000, eBioscience, Waltham, MA, USA) for 30 min at 4°C to determine cell viability. Next, the cells were washed two times with PBS and stained with fluorochrome-labelled antibodies for 30 min at 4°C in 100 *μ*l of staining buffer (PBS with 2% FBS (Sigma-Aldrich)) and 2 mM EDTA (Gibco, Waltham, MA, USA). Fluorochrome-conjugated anti-mouse antibodies used for surface staining included Alexa Fluor 647 CD3*ε* (1 : 200, clone 145-2C11, BioLegend, San Diego, CA, USA), Pacific Blue CD45.2 (1 : 200, clone 104, BioLegend), and PE TCR *γδ* (1 : 400, clone GL3, STEMCELL Technologies, Vancouver, Canada). After incubation, the cells were washed twice in PBS and then fixed in 1% PBS-buffered formaldehyde overnight. Before FACS analysis, samples were washed twice in PBS. Appropriate isotype controls were used to determine specific staining. Compensation beads were used to perform the compensation (UltraComp eBeads, Invitrogen, Waltham, MA, USA). Samples were analyzed using LSRFortessa (BD Biosciences, Franklin Lakes, NJ, USA). The antibody concentrations and gating strategy that we used in this work were described earlier in [11] and were followed exactly the same way. Briefly, an initial gate was applied to include the cells of interest, followed by doublet exclusion. Next, a viability gate was applied to exclude dead cells. Based on the live cell gate, a GFP gate was created which was used for *γδ* T cell number measurements. The identity of GFP cells was confirmed by CD45, CD3, and *γδ* TCR expression. Absolute cell number counts in each sample were determined by CountBright counting beads (Invitrogen, Carlsbad, CA, USA) following the manufacturer's recommendations. Data were analyzed using the FlowJo software (Tree Star Inc., Ashland, OR, USA).

### 2.5. Microscopy Analysis

#### 2.5.1. Tissue Clearing

Microscopy analysis of cleared vaginas from Tcrd-H2BeGFP transgenic mice was done using a previously developed protocol [12]. Isolated vaginas were immediately fixed for 2 h in ice-cold 3.7% (vol/vol) formaldehyde (POCH, Gliwice, Poland) and washed overnight in PBS. Next, the vaginas were kept in the CUBIC-1 reagent diluted 1 : 1 in distilled water for 1 day at 37°C and then transferred to undiluted CUBIC-1 reagent for 2 days at 37°C with gentle shaking. After three washes in PBS at RT with gentle shaking, samples were placed in 30% (*w*/*v*) sucrose in PBS for 1 day and then moved to Tissue-Tek O.C.T. Compound (Sakura, Alphen aan den Rijn, Netherlands) and frozen at -80°C overnight. Samples were then thawed and washed with PBS three times at RT followed by staining with anti-laminin 5 antibody (Abcam, Cambridge, UK, ab14509, dilution 1 : 200) and DAPI (Sigma-Aldrich, 0.5 *μ*g/ml) in BlockAid Blocking Solution (Thermo Fisher Scientific) for 2 days at 37°C. Stained samples were then washed with PBS three times and incubated with the secondary antibody—Alexa Fluor 555-labeled goat anti-rabbit IgG antibody (Thermo Fisher Scientific, A-21429, dilution 1 : 1000) for 1 day at 37°C. After washing in PBS, samples were immersed in the CUBIC-2 reagent diluted 1 : 1 in distilled water for 1 day at 37°C and then in the undiluted CUBIC-2 reagent for 1 day at 37°C. For imaging, samples were mounted in the CUBIC-2 reagent.

#### 2.5.2. Microscopic Imaging

The imaging of whole cleared vaginas was performed on a resonant scanning Leica SP8 system (Leica Microsystems, Wetzlar, Germany) equipped with a femtosecond pulsed Chameleon Vision II (Coherent, Santa Clara, CA, USA) infrared laser and three visible light laser lines (488, 552, and 638 nm). A water immersion 25x (NA 0.95) objective with a cover slip and infrared light correction was used. Collagen was visualized using second harmonic generation (SHG) at 800 nm. GFP in *γδ* T lymphocyte nuclei was excited with the 488 nm line (single-photon imaging) or at 900 nm (2-photon imaging). Laminin 5 was detected with the Alexa Fluor 555-labeled secondary antibody using the 552 nm laser line (single-photon imaging). Samples were imaged in a sequential mode starting with the visible laser lines to avoid photobleaching of Alexa Fluor dyes by the infrared laser. For 2-photon microscopy, internal spectral PMT detectors were used at fully opened pinhole. Single-photon imaging was performed with pinhole set to 1-2 AU.

#### 2.5.3. Image Analysis

Image processing was performed in Imaris (Bitplane, Zurich, Switzerland) and Fiji/ImageJ software (National Institutes of Health, Bethesda, MD, USA). All channels were filtered with a 3 × 3 median filter. Autofluorescence in vaginal samples present in GFP channel (500–550 nm emission range) was masked using a far red range emission channel (650–750 nm) excited with the 638 nm line which contained the same non-GFP structures and diffused background. The number of *γδ* T cells in vaginal compartments was analyzed manually by marking GFP^+^ cells with a paintbrush tool in Fiji/ImageJ (small or big dot in white color for cells in epithelium and stroma, respectively). Channels showing autofluorescence and collagen layer were used to distinguish vaginal epithelium from stroma. Next, the image with dots was binarized to produce an image only with dot-marked *γδ* T cells, which were quantified based on dot size using 3D Objects Counter plugin [13] in Fiji/ImageJ. Vaginal epithelium was rendered for visualization in the Imaris software.

### 2.6. Gene Expression Analysis

Isolated vaginas were stored in 300 *μ*l of FixRNA reagent (EURX, Gdańsk, Poland) at -80°C. RNA was extracted using the DNA/RNA Extracol Kit (EURX) according to the protocol provided by the manufacturer. RNA concentration and purity were assessed using a UV-Vis NanoDrop 8000 spectrophotometer (Thermo Fisher Scientific). Total RNA (1 *μ*g) was used for cDNA synthesis using the RT^2^ First Strand Kit (Qiagen, Hilden, Germany) according to the manufacturer's protocol. The relative expression of 84 aging-related genes was evaluated using the RT^2^ Profiler™ PCR Array Mouse Aging kit (Qiagen) according to the manufacturer's instructions. For analysis, cDNA obtained after isolation and transcription of 1 *μ*g RNA was then mixed with RT^2^ SYBR Green Mastermix reagent and pipetted (25 *μ*l) into a 96-well RT^2^ Profiler PCR plate containing lyophilized primers of 84 genes and 5 housekeeping genes (*Actb*, beta actin; *B2m*, beta-2 microglobulin; *Gapdh*, glyceraldehyde-3-phosphate dehydrogenase; *Gusb*, beta glucuronidase; and *Hsp90ab1*, heat shock protein 90 alpha family class B member 1), one genomic DNA control, 3 reverse-transcription controls, and 3 positive PCR controls. The amplification was performed in a Stratagene Mx3005P thermocycler (Thermo Fisher Scientific). Cycling conditions were 95°C for 10 min, 40 cycles at 95°C for 15 s, and 60°C for 1 min. The raw data (Ct values) obtained as a result of the PCR reaction were imported into the RT^2^ Profiler PCR Array Data Analysis program, which was used to assess the gene expression profile in the studied groups. The background cut-off level was assumed to be 35 (set Ct cut-off = 35). Data were normalized to the arithmetic mean of the *B2m* gene (the lowest standard deviation of the 5 housekeeping genes). The fold regulation (FR) and statistical significance *p* < 0.05 were assumed as the significance limit of changes in the gene expression (i.e., lowering or increasing its level in relation to the control group). This meant that the expression of the gene had to differ at least 2-fold to be considered as deviating from the expression in the control group.

### 2.7. Cytokine Analysis

After isolation, the vaginas and uteri were placed in 500 *μ*l of PBS buffer containing 1.5 mM Pefabloc SC (Sigma-Aldrich), 0.1 mg/ml of soybean trypsin inhibitor (Thermo Fisher Scientific), 0.05 M EDTA (Sigma-Aldrich), and 1% BSA (Sigma-Aldrich). The samples were then frozen at -80°C. The day before the scheduled measurement of the cytokine concentration, samples were thawed and permeabilized overnight in a saponin buffer (2% saponin (*w*/*v*) (Carl Roth, Germany) in PBS solution) at 4°C (the vagina in 200 *μ*l of buffer and the uterus in 400 *μ*l of buffer). The next day, the samples were centrifuged at 15,000 × g for 10 minutes and 25 *μ*l of cytokine-rich supernatant from permeabilized organs was collected for analysis according to manufacturer's protocol of the LEGENDplex ™ Mouse Inflammation Panel kit (BioLegend). Data acquisition was done with the FACS Canto II Flow Cytometer (BD Bioscience, USA). BioLegend's LEGENDplex™ Data Analysis Software was applied for analysis (http://www.biolegend.com/legendplex).

### 2.8. Statistical Analysis

Statistical analysis was performed using the Mann–Whitney *U* test (two-group comparisons) or ANOVA test (four-group comparisons) as detailed in figure captions. The *p* value less than 0.05 was considered statistically significant. All statistical analysis was performed with the GraphPad Prism v6 software (GraphPad Software Inc., La Jolla, CA, USA).

## 3. Results and Discussion

### 3.1. Analysis of *γδ* T Cell Number and Distribution during Aging in the Reproductive Tract

**Figure 1:**
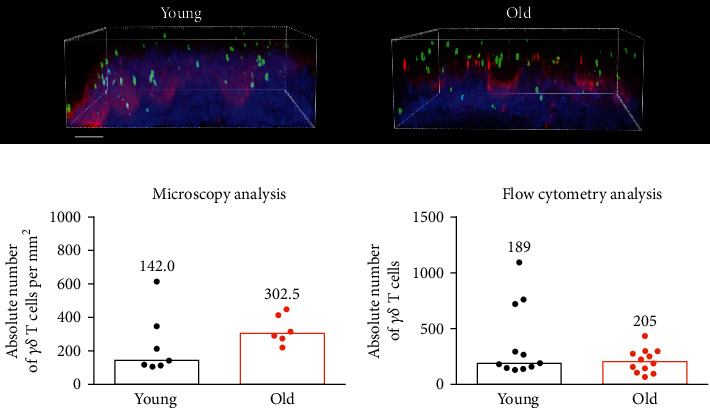
The analysis of vaginal *γδ* T cell numbers in young and old mice. (a) Microscopic visualization of *γδ* T cells in the vaginas of Tcrd-H2BeGFP mice. Side projections of vaginal wall with GFP-expressing *γδ* T cells. GFP shown in green, laminin 5 in red, and stromal collagen in blue. Scale bar = 50 *μ*m. (b, c) Quantification of *γδ* T cells in murine vagina in young and old mice using microscopy (b) and cytometry (c). Each spot represents an individual young (black, 2 months old) and old mouse (orange, 18 months old). *N* = 7 mice per group (microscopy); *n* = 12 mice per group (cytometry). Bars and numbers show medians. No statistically significant differences between groups (Mann–Whitney *U* test).

The vaginal epithelium populated by *γδ* T cells with the canonical V*γ*6-V*δ*1 TCR plays the most critical role in the protection of the organism from microbial insults through the reproductive tract; thus, we determined the impact of age on the total *γδ* T cell number in the murine vagina to see whether the immune surveillance is maintained over time. In mice, reproductive alterations begin at 13-14 months of age, and by 17 months, approximately 80% of female mice cycle irregularly or are acyclic [14]. Vaginal staging of the estrous cycle revealed persistent diestrus in all analyzed 18-month-old animals (data not shown). As a control, we used 2-month-old mice with hormonally induced diestrus phase. For the quantification of *γδ* T cells in the murine vagina during the lifespan, we used our previously developed microscopy [12] and flow cytometry [11] protocols. The number of vaginal *γδ* T cells was stable with aging, as determined with both utilized methods (Figure 1). Microscopic analysis showed a small increase in *γδ* T lymphocyte number; however, it did not reach the statistical significance. By employing enzymatic digestion and flow cytometry analysis, we typically obtained about 200-400 *γδ* T cells from a single vagina regardless of age.

**Figure 2:**
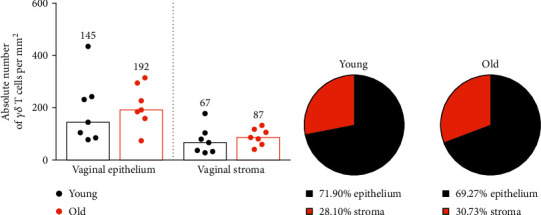
Analysis of *γδ* T cell distribution in vaginal compartments in young and old mice. (a) Absolute number of *γδ* T cells in vaginal epithelium and stroma based on microscopic image analysis. Each spot represents an individual young mouse (black, 2 months old) or old mouse (orange, 18 months old). *N* = 7 mice per group. Bars and numbers show medians. No statistically significant differences between groups (Mann–Whitney *U* test). (b) The percentage distribution of *γδ* T cells in vaginal compartments in young and old mice.

Next, we checked whether the distribution of *γδ* T cells in the vaginal epithelium and stroma changes during aging. As we previously showed in the diestrus phase, the ratio of epithelial versus stromal *γδ* T cell numbers was constant, close to 3 : 1 [12]. In old females, the *γδ* T cell number in both vaginal compartments remained similar compared to young females (Figure 2(a)). There were 2-3 times more GFP^+^*γδ* T cells in the vaginal epithelium in comparison to the vaginal stroma, regardless of age (Figure 2(b)).

Since aged female mice cease to reproduce due to hormonal changes, the uterus is no longer capable of supporting pregnancy. We wondered whether the age-related hormonal status may also affect the quantity of uterine *γδ* T lymphocytes, which can be a more heterogeneous group of cells based on their TCR repertoire [15]. To our surprise, mice at the age of 18 months had a twofold increase in the *γδ* T cell number in the uterus (Supplementary Figure 1). Interestingly, Monin et al. [16] found that in 16-week-old mice, there was a decrease in the representation of *γδ* T cells among all uterine CD3^+^ T cells compared to 4-week-old females; however, the absolute numbers of *γδ* T lymphocytes were not demonstrated.

Overall, these data indicate that the population of *γδ* T cells in the vagina remains persistent, at least over the period of 18 months. It is analogous to dendritic epidermal T cells (DETCs), a V*γ*5^+^*γδ* T cell population present in the skin, which has a self-renewing potential and keeps its number stable over the mouse lifespan [17]. The limiting factor of our studies is the identity of *γδ* T cells that were analyzed. Our microscopic analyses did not include V*γ*5^+^*γδ* T lymphocytes as they are present only at the vaginal opening, shown in our previous work [12]. Still, it is possible that other than V*γ*6^+^*γδ* T lymphocytes (i.e., with V*γ*1 chain or V*γ*2 instead of V*γ*6) migrate from the periphery to the vagina or uterus, which could explain the increase of *γδ* T cell number in the latter location. Alternatively, local proliferation of V*γ*6^+^*γδ* T cells within the uterus is possible in response to age-related changes.

### 3.2. Analysis of the Expression of Age-Related Genes in Vaginal Tissues

In mice lacking *γδ* T cells, a replacement *αβ* T lymphocyte population exists in the epidermis, which disappears with age diminishing the immune surveillance of the skin [17]. Thus, we decided to analyze vaginal tissues of young and old females isolated from WT and *γδ* T-cell-deficient (*Tcrd*^−/−^) mouse strains in search of differences indicating the disturbance of tissue homeostasis in aged FRT in the absence of *γδ* T cells. We focused on the quantification of gene expression of 84 genes associated with metabolic and cellular changes during aging [18–20]. The list of analyzed genes is provided in Supplementary Table 1. After performing the analysis, we did not observe any statistically significant differences in the gene expression between young mice of both strains (Table 1 and Supplementary Figure 2).

Surprisingly, the vaginas from old WT and *Tcrd*^−/−^ mice differed only in one gene, *Ltf*, which encodes lactotransferrin (Ltf) protein. We found that *Ltf* was decreased in aged *Tcrd*^−/−^ mice. Ltf, also known as lactoferrin, is an iron-binding glycoprotein present in milk, saliva, and other exocrine secretions [21]. Ltf has multiple biological functions, including antimicrobial and immunomodulatory effects. The Ltf level rises with the increase of estradiol concentration in blood [22] and also during genital infections upon release from neutrophils infiltrating the infection site [23, 24]. In addition, all major lymphocyte subsets produce Ltf after activation. Importantly, the proportion of Ltf-expressing activated *γδ* T cells is significantly larger than that of activated *αβ* T cells [25]. Thus, the lack of *γδ* T cells may be responsible for the decreased *Ltf* expression in aged *Tcrd*^−/−^ mice, potentially leading to weaker antimicrobial responses in the absence of *γδ* T cells.

We have also found that the *S100a9* gene expression was decreased with age in the WT strain (Table 1 and Supplementary Figure 2). When comparing old WT to old *Tcrd*^−/−^ mice, we noticed a further reduction in this gene expression in mice lacking *γδ* T cells; however, it did not reach the statistical significance. S100a9 (calgranulin B) is a small molecular weight calcium-binding protein of the S100 family [26]. S100a9 elicits antimicrobial properties to various microbial pathogens, including *C. albicans* [27]. Together with S100a8 (calgranulin A), S100a9 decreases the adaptive T cell immune responses by preventing the differentiation and development of DCs. Moreover, S100a9 is able to promote IL-8 secretion and a degranulation process by human neutrophils as well as proinflammatory cytokine production by monocytes and macrophages [28]. Based on *S100a9* and *Ltf* gene expression analyses, we conclude that the mechanisms of antimicrobial responses are diminished during aging, which could be further worsened in mice deficient in *γδ* T cells.

The comparison of gene expression in tissues from young and old *Tcrd*^−/−^ mice revealed upregulation of four genes, *C1qb*, *C1qc*, *C3ar1*, and *Clu*, in aged females of this strain (Table 1 and Supplementary Figure 2). When comparing old WT to old *Tcrd*^−/−^ mice, we found a similar trend of increased expression of these genes in the absence of *γδ* T lymphocytes; however, the differences did not reach the statistical significance. The first three genes code proteins belonging to the complement system, which is traditionally considered as a part of innate immunity. C1q is the main protein of the classical complement cascade and is composed of three similar but distinct subunits, i.e., A, B, and C [29]. C3 is the dominant protein of the alternative complement pathway and binds to its receptor C3ar1, encoded by *C3ar1* gene. Besides the engagement in the complement activity, these proteins also induce dendritic cell maturation and modulate the subsequent development of effector T cell responses [30]. Moreover, enhanced C3ar1 signaling plays a role in the induction of vascular and mucosal inflammation [31, 32]. Interestingly, C3ar1 was shown to be upregulated in different carcinoma subtypes [33]. The complement system activation is also involved in the pathophysiology of pregnancy [34] and endometriosis [35]. The overexpression of the three complement genes in old *Tcrd*^−/−^ mice may indicate a dysregulation of immune responses and inflammation control without *γδ* T cells leading to increased carcinogenesis, reproductive tract dysfunctions, and diminished regeneration capacity in aging individuals [36].

Clusterin (CLU), also known as apolipoprotein J (apoJ), is widely distributed in tissues and body fluids [37]. *Clu* is an estrogen-responsive gene, and in the presence of estrogens, CLU expression in the uterus is decreased [38]. The *Clu* gene overexpression during aging is in line with published human data [39] and our observation in aged female mice, which have low estrogen levels. Recent studies demonstrated that increased CLU production has a strong relationship with gynecological malignancies, i.e., ovarian carcinoma [40] and cervical cancer [41]. Our finding of increased *Clu* expression in old *Tcrd*^−/−^ mice may be in line with a higher susceptibility of this strain to carcinogenesis [42].

Obtained gene expression data do not show any robust effect of *γδ* T cells in the regulation of metabolic and cellular changes in aging vaginal tissue; however, the results require further validation by protein expression analysis as gene expression changes do not necessarily involve protein expression modifications.

### 3.3. Analysis of Inflammatory Cytokines during Aging in the Reproductive Tract

The aging process is characterized by prolonged inflammation which ultimately leads to disturbed tissue homeostasis [43]. We confirmed this phenomenon in FRT by analyzing levels of 13 cytokines that play an important role in the regulation of inflammatory processes, either secreted at the steady state or upon inflammation. To induce inflammation, we used a CpG 1826 oligodeoxynucleotide (ODN), a TLR9 agonist activating both innate and adaptive immune responses. When administered to the vaginal mucosa, CpG causes extensive local inflammation as shown previously by histological analysis [44].

**Figure 3:**
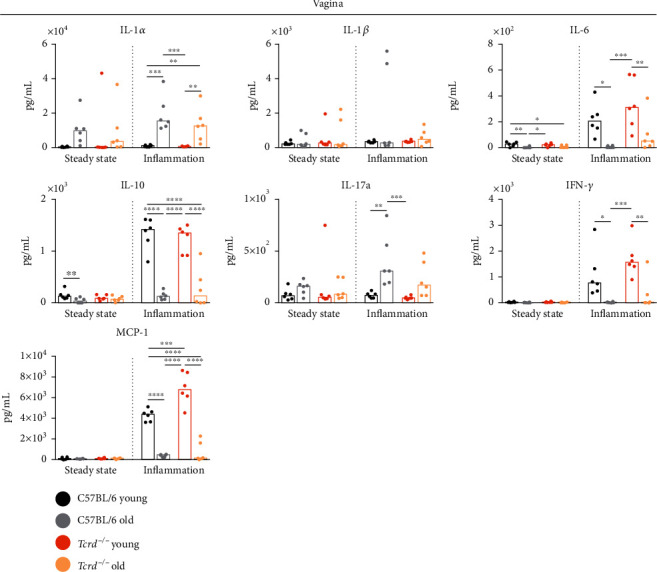
The vaginal cytokine profile at the steady state and upon inflammation. Concentrations of cytokines in the vaginal wall in WT and *Tcrd*^−/−^ mice. Each spot represents an individual mouse; *n* = 6 mice per group; bars show medians; ^∗^*p* < 0.05; ^∗∗^*p* < 0.01; ^∗∗∗^*p* < 0.001; ^∗∗∗∗^*p* < 0.0001, ANOVA test.

The obtained data indicate an intensification of inflammatory processes in the vaginas of old mice, regardless of the presence of *γδ* T cells (Figure 3 and Supplementary Figure 3). This was evidenced by an increase in the production of a proinflammatory IL-1*α* and a decrease in the amount of IL-10, which has anti-inflammatory activity. The expression of IL-1*α* is increased during infection and inflammation [45]. IL-1*α* is an “alarmin” that acts locally, while IL-1*β* is secreted into the blood and has a systemic effect [45]. Neither age nor the absence of *γδ* T cells did not affect IL-1*β* concentration in examined tissues. IL-17A, another proinflammatory cytokine, was reported to be produced within the FRT mainly by *γδ* T lymphocytes [46, 47]. Our data partially corroborated these findings as only in aged WT mice, there was a statistically significant increase of IL-17A level in response to CpG stimulation, possibly due to presence of *γδ* T cells. The vaginal production of IL-6, which shows both pro- and anti-inflammatory effects, depending on the microenvironmental context, was inhibited in old mice of both strains upon inflammation, which further adds to a progressive dysregulation of immune responses with age. Additionally, the measurement of IFN-*γ* concentrations demonstrated lower levels in the inflamed tissues of old mice, indicating that Th1 cell polarization, antiviral resistance, and antigen presentation processes may be less effective in old age. The changes in IL-1*α*, IL-10, IL-17A, and IFN-*γ* levels under the inflammatory state had an analogous trend in the uteri of aged females (Supplementary Figure 4).

Similarly to IFN-*γ* production, the aging process within the vagina affected MCP-1 protein, which recruits monocytes, memory T cells, and DCs to the sites of inflammation (Figure 3). In the inflamed vaginal tissues of old individuals, we found a significant reduction in the production of this cytokine. It may suggest a lower influx of cells responsible for anti-infectious immunity to the aged epithelium. Interestingly, we observed an intensified MCP-1 release in the vaginas of young *Tcrd*^−/−^ mice compared to WT mice. This effect could indicate an inhibitory role of *γδ* T cells in stimulating this cytokine or the activation of other mechanisms dependent on surrogate cells that may appear in the vaginal epithelium, similar to *αβ* T lymphocytes in the epidermis of *Tcrd*^−/−^ mice [17]. A different pattern of MCP-1 production was noticed in the uteri (Supplementary Figure 4). In WT mice, MCP-1 level increased with age at the steady state and was higher than in *γδ* T cell-deficient mice. Upon inflammation in the absence of *γδ* T lymphocytes, MCP-1 concentration was diminished in aged females, when compared to old WT mice and young *Tcrd*^−/−^ mice. The differences between vaginal and uterine tissues with regard to *γδ* T cell-regulated MCP-1 production may result from a different cellular composition of both compartments and involvement of other *γδ* T cell subsets that can reside in the uteri and have other functions [47].

## 4. Conclusions

In summary, we confirmed that the aging process affects immune responses within FRT, shifting them toward a more proinflammatory type. The data coming from the comparisons of WT to *Tcrd^−/−^* mice do not show any robust effect of *γδ* T lymphocytes in controlling the age-related inflammation. *Γδ* T lymphocytes seem to play rather a fine-tuning role in the regulation of tissue homeostasis. In their absence, it is likely that other immune cell populations take over the epithelial homeostasis maintenance. Our results indicate that intraepithelial *γδ* T lymphocytes may contribute to innate antimicrobial responses and modulate functions of other immune cells, depending on the FRT compartment and cellular context of the tissue. Future investigations are still needed to determine the identity of FRT-resident *γδ* T cells based on the TCR composition and their functions in age-related changes in analyzed tissues.

## Figures and Tables

**Table 1:** Summary of the gene expression analysis of genes associated with the aging process in the vaginas of WT and *Tcrd*^−/−^ mice. FR: fold regulation; *p*: *p* value. Genes for which FR > 2 and *p* < 0.05 or FR < 2 and *p* < 0.05 are in italic. The analysis performed on *n* = 3 mice per group.

Gene symbol	Unigene	Gene description	Function	C57BL/6 young vs. *Tcrd^−/−^* young		C57BL/6 old vs. *Tcrd^−/−^* old		C57BL/6 young vs. C57BL/6 old		*Tcrd^−/−^* young vs. *Tcrd^−/−^* old	
				FR	*p*	FR	*p*	FR	*p*	FR	*p*
*C1qb*	Mm.2570	Complement component 1, q subcomponent, beta polypeptide	Inflammatory response	-1.91	0.401	38.23	0.371	-17.43	0.372	*4.20*	*0.048*
*C1qc*	Mm.439732	Complement component 1, q subcomponent, C chain	Inflammatory response	-1.53	0.271	5.22	0.058	-2.04	0.185	*3.91*	*0.014*
*C3ar1*	Mm.2408	Complement component 3a receptor 1	Inflammatory response	-1.95	0.199	45.57	0.374	-30.55	0.374	*2.91*	*0.012*
*Clu*	Mm.200608	Clusterin	Apoptosis	1.06	0.833	2.53	0.316	1.74	0.385	*4.15*	*0.047*
*Ltf*	Mm.282359	Lactotransferrin	Inflammatory response	-1.53	0.298	*-4.33*	*0.009*	1.96	0.333	-1.44	0.221
*S100a9*	Mm.2128	S100 calcium binding protein A9 (calgranulin B)	Inflammatory response	-1.63	0.421	-8.51	0.182	*-7.96*	*0.045*	-41.55	0.166

## Data Availability

The original data used to support the findings of this study are available from the corresponding author upon request.

## Supplementary Materials

Supplementary Materials Supplementary Material.docx (Figures S1 – S4 and Table S1).
