# Supplementary material for: Age-Related Changes in Female Murine Reproductive Mucosa with respect to γδ T Cell Presence
Source: J Immunol Res. 2023 Jan 23;2023:3072573. doi: 10.1155/2023/3072573 (PMC9886474; doi:10.1155/2023/3072573)

**Supplementary Materials**

**Age-related changes in female murine reproductive mucosa with respect to γδ T cell presence**

*Journal of Immunology Research*

Katarzyna Skulska^1,2^, Anna Kędzierska^1,2^, Małgorzata Krzyżowska^1,3^, Grzegorz Chodaczek^1#^

^1^ Łukasiewicz Research Network – PORT Polish Center For Technology Development, Wroclaw, Poland

^2^ Hirszfeld Institute of Immunology and Experimental Therapy, Polish Academy of Sciences, Wroclaw, Poland

^3^ Military Institute of Hygiene and Epidemiology, Warsaw, Poland

# Corresponding author: [grzegorz.chodaczek@port.lukasiewicz.gov.pl](mailto:grzegorz.chodaczek@port.lukasiewicz.gov.pl)


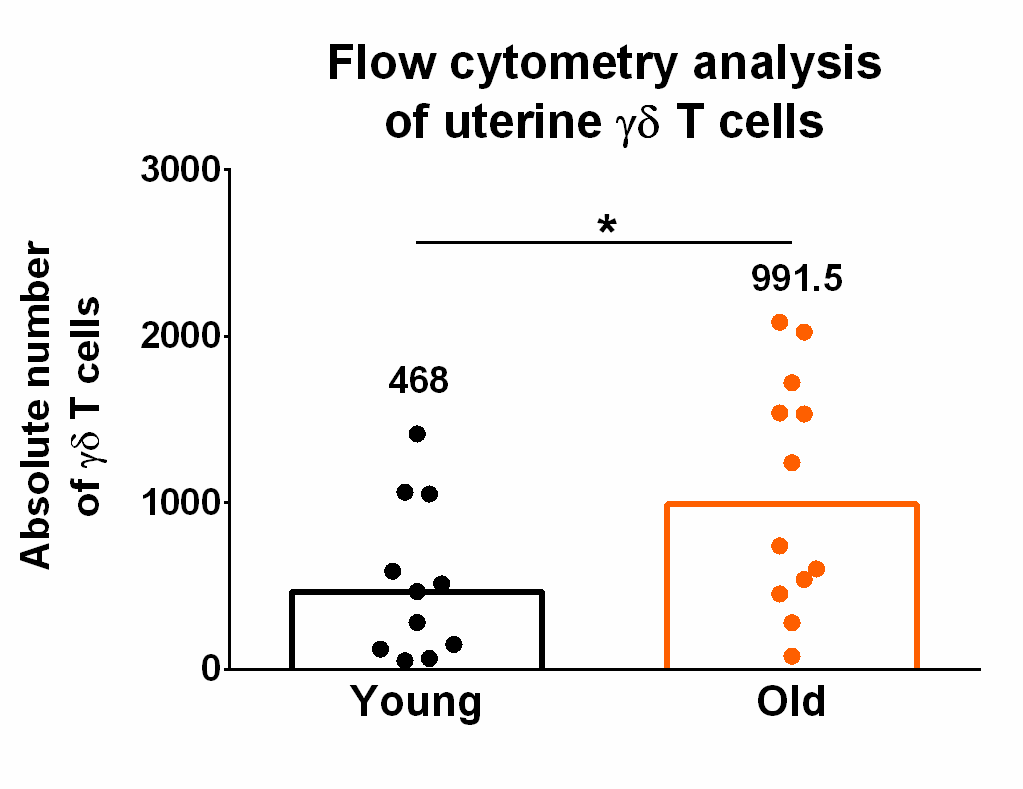


**Supplementary Fig. 1** Quantification of γδ T cells in murine uterus in young and old mice. Flow cytometry analysis of uterine γδ T cells from C57BL/6J mice. Each spot represents an individual young (black, 2 months old) and old mouse (orange, 18 months old). N = 12 mice per group. Bars and numbers show medians. **p*<0.05 (Mann-Whitney U test)

**Supplementary Table 1** Analysis of the expression of genes regulating the aging process in vaginas of WT and *Tcrd*^-/-^ mice. Alphabetical order. FR – fold regulation, *p* – *p*-value. Genes for which FR>2 and *p*<0.05 are marked in red. Genes for which FR<-2 and *p*<0.05 are marked in blue.

| **Gene symbol** | **C57BL/6 young vs. C57BL/6 old** | | **C57BL/6 young vs.**  ***Tcrd^-/-^* young** | | **C57BL/6 young vs. *Tcrd^-/-^* old** | | **C57BL/6 old vs. *Tcrd^-/-^* young** | | **C57BL/6 old vs. *Tcrd^-/-^* old** | | ***Tcrd^-/-^* young vs. *Tcrd^-/-^* old** | |
| --- | --- | --- | --- | --- | --- | --- | --- | --- | --- | --- | --- | --- |
|  | **FR** | ***p*** | **FR** | ***p*** | **FR** | ***p*** | **FR** | ***p*** | **FR** | ***p*** | **FR** | ***p*** |
| ***Angel2*** | -49,64 | 0,374 | -4,08 | 0,745 | -3,05 | 0,791 | 12,15 | 0,374 | 16,30 | 0,374 | 1,34 | 0,992 |
| ***Anxa3*** | -8,48 | 0,370 | -1,03 | 0,829 | 1,52 | 0,667 | 8,21 | 0,371 | 12,85 | 0,372 | 1,57 | 0,720 |
| ***Anxa5*** | -6,09 | 0,368 | 1,15 | 0,625 | 1,89 | 0,435 | 6,98 | 0,368 | 11,50 | 0,366 | 1,65 | 0,634 |
| ***Arid1a*** | -7,21 | 0,370 | 1,13 | 0,973 | -1,32 | 0,176 | 8,13 | 0,370 | 5,45 | 0,372 | -1,49 | 0,422 |
| ***Arl6ip6*** | -7,19 | 0,369 | 1,27 | 0,608 | -1,59 | 0,142 | 9,17 | 0,368 | 4,53 | 0,372 | -2,02 | 0,136 |
| ***Bub1b*** | -12,15 | 0,374 | -1,01 | 0,734 | 1,24 | 0,633 | 11,99 | 0,374 | 15,03 | 0,374 | 1,25 | 0,575 |
| ***C1qa*** | 2,51 | 0,803 | -1,26 | 0,436 | 3,00 | 0,160 | -3,16 | 0,956 | 1,19 | 0,400 | 3,78 | 0,077 |
| ***C1qb*** | -17,43 | 0,372 | -1,91 | 0,401 | 2,19 | 0,239 | 9,11 | 0,373 | 38,23 | 0,371 | **4,20** | **0,048** |
| ***C1qc*** | -2,04 | 0,185 | -1,53 | 0,271 | 2,56 | 0,083 | 1,33 | 0,384 | 5,22 | 0,058 | **3,91** | **0,014** |
| ***C1s1*** | -21,91 | 0,368 | -1,55 | 0,148 | -1,25 | 0,482 | 14,16 | 0,369 | 17,47 | 0,370 | 1,23 | 0,769 |
| ***C3*** | -3,65 | 0,376 | -6,70 | 0,142 | -4,03 | 0,578 | -1,84 | 0,388 | -1,10 | 0,382 | 1,66 | 0,474 |
| ***C3ar1*** | -30,55 | 0,374 | -1,95 | 0,199 | 1,49 | 0,281 | 15,63 | 0,374 | 45,57 | 0,374 | **2,91** | **0,012** |
| ***C4a*** | -5,76 | 0,376 | -1,04 | 0,700 | -1,79 | 0,458 | 5,54 | 0,375 | 3,23 | 0,381 | -1,72 | 0,370 |
| ***C4b*** | -25,99 | 0,324 | -1,64 | 0,317 | -1,76 | 0,433 | 15,89 | 0,326 | 14,76 | 0,330 | -1,08 | 0,605 |
| ***C5ar1*** | -6,35 | 0,369 | -2,61 | 0,276 | -3,82 | 0,077 | 2,43 | 0,403 | 1,66 | 0,407 | -1,46 | 0,892 |
| ***Calb1*** | -9,92 | 0,373 | 1,19 | 0,866 | -4,39 | 0,208 | 11,82 | 0,373 | 2,26 | 0,383 | -5,23 | 0,205 |
| ***Casp1*** | -2,14 | 0,280 | -1,06 | 0,884 | -4,70 | 0,258 | 2,02 | 0,293 | -2,20 | 0,425 | -4,45 | 0,262 |
| ***Ccr1*** | -4,25 | 0,372 | -1,47 | 0,414 | -3,45 | 0,121 | 2,88 | 0,412 | 1,23 | 0,457 | -2,34 | 0,514 |
| ***Cd14*** | -3,10 | 0,584 | -1,06 | 0,399 | -7,53 | 0,134 | 2,91 | 0,645 | -2,43 | 0,250 | -7,08 | 0,531 |
| ***Cd163*** | -20,68 | 0,370 | -1,37 | 0,146 | -1,82 | 0,231 | 15,10 | 0,370 | 11,39 | 0,371 | -1,33 | 0,402 |
| ***Cdkn1c*** | -5,54 | 0,369 | -1,01 | 0,835 | **2,83** | **0,036** | 5,48 | 0,370 | 15,71 | 0,360 | 2,87 | 0,100 |
| ***Cfh*** | -3,68 | 0,089 | -1,55 | 0,131 | -1,19 | 0,562 | 2,38 | 0,132 | 3,08 | 0,105 | 1,30 | 0,356 |
| ***Cfhr1*** | -7,18 | 0,372 | 1,19 | 0,866 | -4,39 | 0,208 | 8,55 | 0,371 | 1,64 | 0,400 | -5,23 | 0,205 |
| ***Clu*** | 1,74 | 0,385 | 1,06 | 0,833 | **4,41** | **0,031** | -1,64 | 0,471 | 2,53 | 0,316 | **4,15** | **0,047** |
| ***Cx3cl1*** | -2,89 | 0,383 | -1,33 | 0,538 | 2,08 | 0,134 | 2,17 | 0,389 | 6,02 | 0,372 | 2,77 | 0,111 |
| ***Cxcl16*** | -1,36 | 0,592 | -1,56 | 0,357 | -1,10 | 0,743 | -1,15 | 0,802 | 1,23 | 0,921 | 1,41 | 0,763 |
| ***Elavl1*** | -3,11 | 0,242 | -1,14 | 0,636 | 1,01 | 0,626 | 2,73 | 0,260 | 3,15 | 0,285 | 1,15 | 0,817 |
| ***Elp3*** | -2,91 | 0,259 | 1,11 | 0,999 | 1,94 | 0,842 | 3,25 | 0,261 | 5,66 | 0,254 | 1,74 | 0,872 |
| ***Eml1*** | -1,82 | 0,339 | 3,56 | 0,271 | 2,04 | 0,250 | 6,47 | 0,284 | 3,71 | 0,288 | -1,74 | 0,930 |
| ***Ep300*** | -3,04 | 0,322 | 1,41 | 0,704 | 1,64 | 0,917 | 4,30 | 0,313 | 4,97 | 0,328 | 1,16 | 0,760 |
| ***Fbxl16*** | -19,93 | 0,366 | 1,22 | 0,915 | -3,30 | 0,216 | 24,36 | 0,366 | 6,03 | 0,371 | -4,04 | 0,213 |
| ***Fcer1g*** | -8,17 | 0,371 | -1,16 | 0,610 | -1,72 | 0,254 | 7,01 | 0,372 | 4,75 | 0,374 | -1,48 | 0,417 |
| ***Fcgbp*** | 2,78 | 0,727 | 1,29 | 0,480 | 8,24 | 0,122 | -2,16 | 0,602 | 2,96 | 0,427 | 6,41 | 0,134 |
| ***Fcgr1*** | -7,80 | 0,373 | -1,15 | 0,632 | 1,47 | 0,437 | 6,79 | 0,373 | 11,45 | 0,372 | 1,69 | 0,277 |
| ***Fcgr2b*** | 1,09 | 0,421 | -1,61 | 0,099 | -1,02 | 0,686 | -1,75 | 0,491 | -1,11 | 0,465 | 1,57 | 0,747 |
| ***Fcgr3*** | -2,99 | 0,154 | -1,40 | 0,300 | -1,24 | 0,392 | 2,14 | 0,241 | 2,41 | 0,266 | 1,13 | 0,934 |
| ***Foxo1*** | -2,56 | 0,323 | 1,01 | 0,907 | -1,01 | 0,643 | 2,59 | 0,327 | 2,54 | 0,365 | -1,02 | 0,686 |
| ***Gfap*** | -4,94 | 0,310 | 1,10 | 0,936 | -1,82 | 0,328 | 5,43 | 0,312 | 2,72 | 0,348 | -2,00 | 0,388 |
| ***Gsta1*** | -3,01 | 0,150 | 2,44 | 0,090 | -2,96 | 0,212 | 7,34 | 0,126 | 1,02 | 0,353 | -7,23 | 0,140 |
| ***Hsf1*** | -4,55 | 0,334 | 1,03 | 0,933 | 1,82 | 0,944 | 4,68 | 0,334 | 8,26 | 0,333 | 1,77 | 0,917 |
| ***Jakmip3*** | -20,49 | 0,373 | 1,19 | 0,866 | -4,39 | 0,208 | 24,42 | 0,373 | 4,67 | 0,375 | -5,23 | 0,205 |
| ***Lmna*** | -2,04 | 0,200 | 1,63 | 0,168 | 4,18 | 0,990 | 3,32 | 0,167 | 8,51 | 0,218 | 2,57 | 0,676 |
| ***Lmnb1*** | -1,10 | 0,474 | 1,95 | 0,739 | 2,10 | 0,734 | 2,15 | 0,432 | 2,31 | 0,431 | 1,08 | 0,993 |
| ***Lmnb2*** | -9,19 | 0,370 | 1,05 | 0,966 | -4,32 | 0,210 | 9,65 | 0,370 | 2,13 | 0,390 | -4,53 | 0,212 |
| ***Lsm5*** | -1,29 | 0,371 | 1,40 | 0,127 | -3,21 | 0,346 | 1,80 | 0,213 | -2,50 | 0,402 | -4,50 | 0,325 |
| ***Ltf*** | 1,96 | 0,333 | -1,53 | 0,298 | **-2,20** | **0,029** | -3,00 | 0,098 | **-4,33** | **0,009** | -1,44 | 0,221 |
| ***Mbp*** | -1,79 | 0,284 | 1,38 | 0,230 | 1,83 | 0,823 | 2,47 | 0,251 | 3,28 | 0,276 | 1,33 | 0,798 |
| ***Mrpl43*** | -3,68 | 0,375 | 1,01 | 0,959 | 1,19 | 0,640 | 3,71 | 0,375 | 4,38 | 0,382 | 1,18 | 0,652 |
| ***Ndufb11*** | -1,29 | 0,415 | 1,09 | 0,613 | -1,75 | 0,061 | 1,40 | 0,347 | -1,36 | 0,942 | -1,90 | 0,027 |
| ***Panx1*** | -3,61 | 0,241 | 1,04 | 0,981 | 1,54 | 0,975 | 3,73 | 0,242 | 5,55 | 0,247 | 1,49 | 0,984 |
| ***Pdcd6*** | -1,36 | 0,428 | 1,30 | 0,491 | -1,11 | 0,583 | 1,76 | 0,359 | 1,22 | 0,692 | -1,44 | 0,453 |
| ***Phf3*** | -4,06 | 0,340 | 1,01 | 0,874 | -1,00 | 0,657 | 4,10 | 0,342 | 4,06 | 0,350 | -1,01 | 0,736 |
| ***Polrmt*** | 1,23 | 0,582 | 1,12 | 0,851 | 1,15 | 0,907 | -1,10 | 0,564 | -1,07 | 0,620 | 1,03 | 0,836 |
| ***Pot1a*** | 1,80 | 0,437 | 1,16 | 0,788 | 1,62 | 0,988 | -1,56 | 0,430 | -1,11 | 0,439 | 1,40 | 0,887 |
| ***Rap1a*** | -3,43 | 0,313 | 1,22 | 0,437 | -1,82 | 0,181 | 4,20 | 0,304 | 1,89 | 0,372 | -2,22 | 0,135 |
| ***Rnf144b*** | 2,20 | 0,612 | 1,69 | 0,181 | 1,18 | 0,565 | -1,30 | 0,489 | -1,87 | 0,836 | -1,43 | 0,354 |
| ***S100a8*** | -7,08 | 0,277 | -2,00 | 0,386 | -133,13 | 0,150 | 3,53 | 0,410 | -18,81 | 0,169 | -66,41 | 0,155 |
| ***S100a9*** | **-7,96** | **0,045** | -1,63 | 0,421 | -67,81 | 0,156 | 4,88 | 0,322 | -8,51 | 0,182 | -41,55 | 0,166 |
| ***Scn2b*** | -4,51 | 0,167 | -1,02 | 0,946 | -2,06 | 0,233 | 4,42 | 0,163 | 2,19 | 0,217 | -2,01 | 0,142 |
| ***Sirt1*** | -3,87 | 0,297 | 1,14 | 0,544 | 1,28 | 0,995 | 4,41 | 0,291 | 4,96 | 0,298 | 1,13 | 0,808 |
| ***Sirt3*** | -2,80 | 0,327 | -1,06 | 0,707 | 1,79 | 0,168 | 2,65 | 0,337 | 5,00 | 0,301 | 1,89 | 0,239 |
| ***Sirt6*** | -4,15 | 0,316 | -1,00 | 0,987 | 1,17 | 0,881 | 4,14 | 0,316 | 4,87 | 0,320 | 1,18 | 0,875 |
| ***Smad2*** | -1,03 | 0,558 | 1,33 | 0,557 | 1,11 | 0,742 | 1,37 | 0,496 | 1,14 | 0,672 | -1,19 | 0,582 |
| ***Snap23*** | -1,56 | 0,293 | -1,08 | 0,648 | -1,08 | 0,590 | 1,45 | 0,347 | 1,45 | 0,557 | -1,00 | 0,708 |
| ***Terf1*** | 1,69 | 0,881 | 1,11 | 0,908 | -1,07 | 0,702 | -1,53 | 0,856 | -1,82 | 0,997 | -1,19 | 0,703 |
| ***Terf2*** | 1,08 | 0,628 | 1,07 | 0,969 | 1,69 | 0,873 | -1,01 | 0,645 | 1,57 | 0,611 | 1,58 | 0,875 |
| ***Tfam*** | -1,99 | 0,198 | 1,10 | 0,891 | -1,37 | 0,260 | 2,18 | 0,196 | 1,45 | 0,315 | -1,51 | 0,300 |
| ***Tfb1m*** | -2,80 | 0,239 | -1,00 | 0,823 | 1,32 | 0,905 | 2,78 | 0,246 | 3,69 | 0,237 | 1,33 | 0,815 |
| ***Tfb2m*** | -2,96 | 0,213 | 1,09 | 0,950 | 1,75 | 0,854 | 3,21 | 0,213 | 5,18 | 0,236 | 1,61 | 0,845 |
| ***Tinf2*** | -2,14 | 0,232 | 1,08 | 0,951 | 1,88 | 0,681 | 2,32 | 0,232 | 4,03 | 0,214 | 1,74 | 0,744 |
| ***Tlr2*** | -18,64 | 0,345 | -7,46 | 0,201 | -7,18 | 0,156 | 2,50 | 0,389 | 2,60 | 0,393 | 1,04 | 0,919 |
| ***Tlr4*** | -1,07 | 0,633 | 1,08 | 0,967 | 1,32 | 0,694 | 1,16 | 0,638 | 1,41 | 0,951 | 1,22 | 0,697 |
| ***Tmem135*** | -4,52 | 0,345 | 1,34 | 0,559 | 2,03 | 0,701 | 6,08 | 0,340 | 9,17 | 0,340 | 1,51 | 0,995 |
| ***Tmem33*** | -1,18 | 0,506 | -1,09 | 0,681 | -2,95 | 0,268 | 1,09 | 0,558 | -2,50 | 0,512 | -2,71 | 0,286 |
| ***Tollip*** | -4,43 | 0,327 | 1,33 | 0,503 | 1,98 | 0,922 | 5,90 | 0,322 | 8,77 | 0,329 | 1,49 | 0,733 |
| ***Tpp1*** | -1,31 | 0,471 | -1,03 | 0,792 | 2,10 | 0,972 | 1,27 | 0,510 | 2,74 | 0,508 | 2,15 | 0,924 |
| ***Txnip*** | 2,03 | 0,776 | -1,05 | 0,747 | 2,11 | 0,362 | -2,13 | 0,667 | 1,04 | 0,822 | 2,22 | 0,381 |
| ***Vps13c*** | -1,68 | 0,240 | -1,06 | 0,728 | 1,32 | 0,679 | 1,58 | 0,345 | 2,21 | 0,211 | 1,40 | 0,576 |
| ***Vwa5a*** | -1,46 | 0,445 | -1,15 | 0,561 | 1,23 | 0,749 | 1,27 | 0,542 | 1,80 | 0,571 | 1,42 | 0,996 |
| ***Wrn*** | -4,59 | 0,346 | -1,04 | 0,751 | 1,51 | 0,883 | 4,44 | 0,348 | 6,93 | 0,344 | 1,56 | 0,759 |
| ***Zbtb10*** | 1,02 | 0,562 | -1,25 | 0,449 | -1,17 | 0,578 | -1,27 | 0,678 | -1,19 | 0,649 | 1,06 | 0,869 |
| ***Zfp9*** | -1,37 | 0,436 | 1,02 | 0,936 | 2,08 | 0,344 | 1,39 | 0,428 | 2,85 | 0,312 | 2,04 | 0,367 |
| ***Zfr*** | -2,10 | 0,280 | 1,19 | 0,550 | -2,92 | 0,333 | 2,50 | 0,250 | -1,39 | 0,604 | -3,47 | 0,319 |
| ***Zmpste24*** | -1,28 | 0,495 | -1,19 | 0,572 | -3,96 | 0,292 | 1,08 | 0,561 | -3,10 | 0,425 | -3,33 | 0,307 |


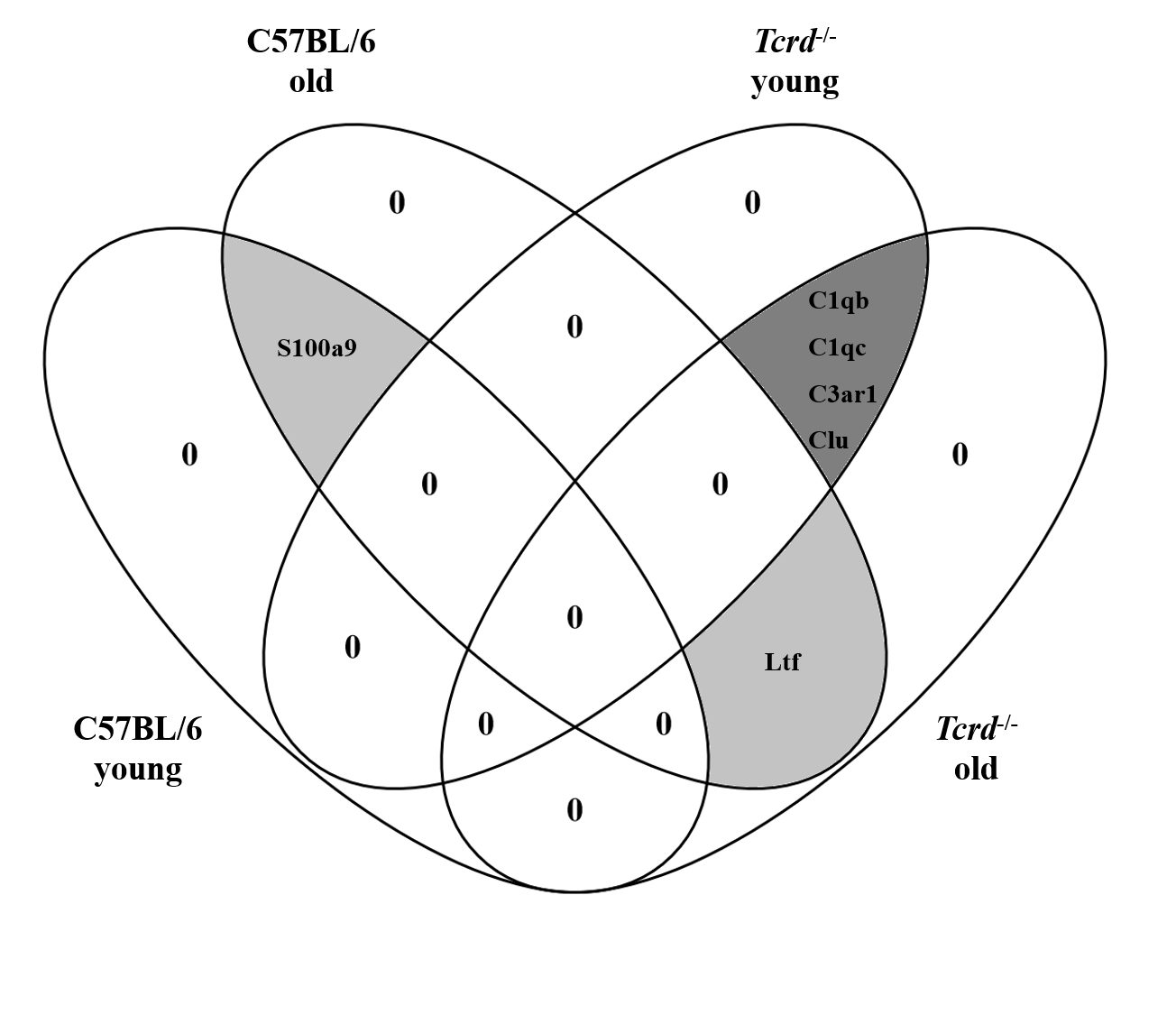


**Supplementary Fig. 2** Venn’s diagram showing the relationship between the expression of genes regulating the aging process in vaginas of WT and *Tcrd*^-/-^ mice. Developed using <https://bioinfogp.cnb.csic.es/tools/venny/index.html> (Oliveros, J.C. (2007-2015) Venny. An interactive tool for comparing lists with Venn's diagrams).


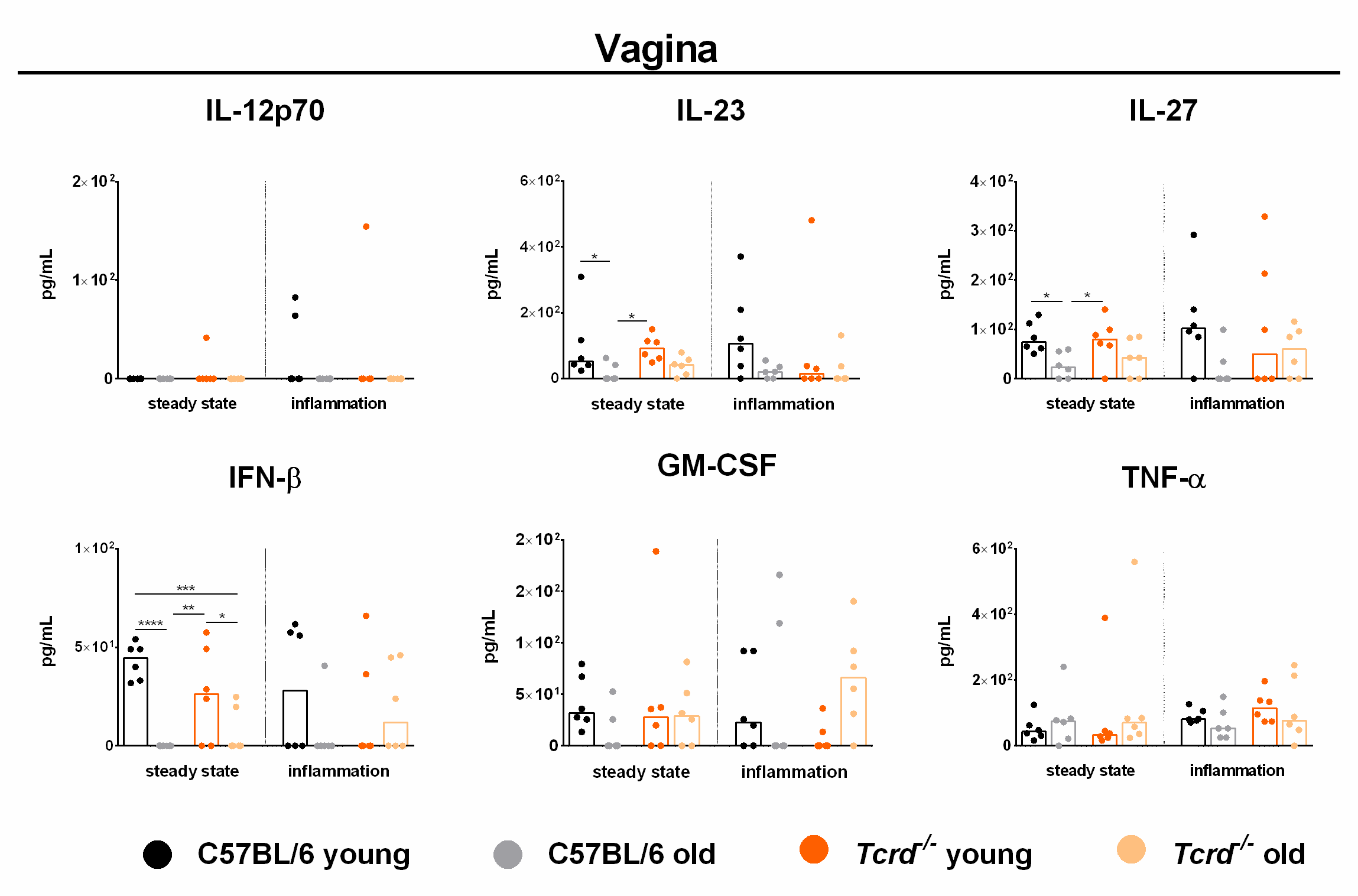


**Supplementary Fig. 3** The vaginal cytokine profile. Concentration of cytokines in the vagina wall at the steady state and upon inflammation in WT and *Tcrd^-/-^* mice. Each spot represents an individual mouse; n = 6 mice per group; bars show medians, *p<0.05; **p<0.01; ***p<0.001; ****p<0.0001, ANOVA test.

**Supplementary Fig. 4** The uterine cytokine profile. Concentration of cytokines in the uterus at steady state and upon inflammation in WT and *Tcrd*^-/-^ mice. Each spot represents an individual mouse; n = 6 mice per group; bars show medians; **p*<0.05; ***p*<0.01; ****p*<0.001; ANOVA test**.**


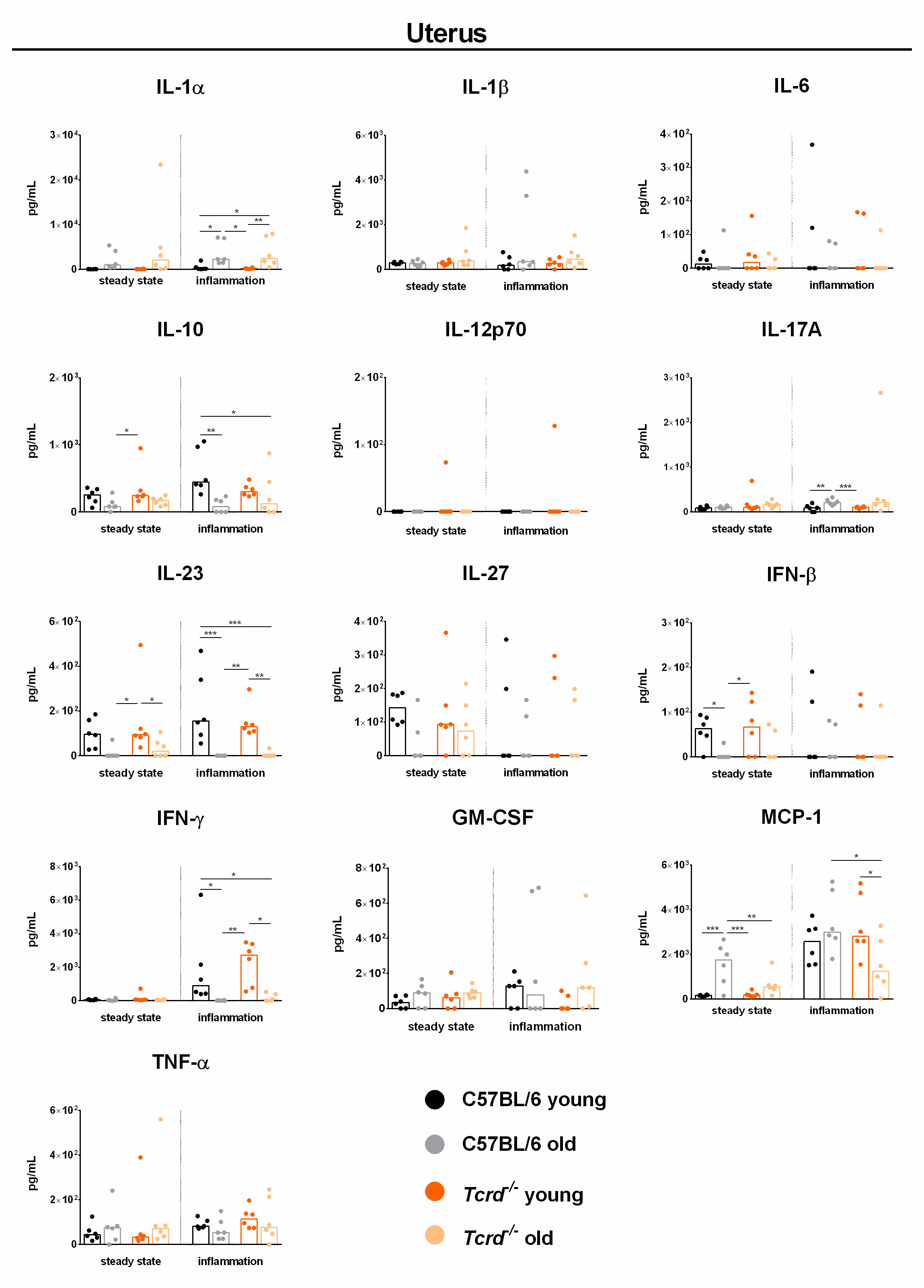

Supplement: Supplementary Materials — Supplementary Material.docx (Figures S1 – S4 and Table S1). [file 3072573.f1.docx]
